# Poly[diaqua­tris­(μ_4_-1,3-phenyl­enediacetato)­dineodymium(III)]

**DOI:** 10.1107/S1600536811006817

**Published:** 2011-02-26

**Authors:** Zhu-Qing Gao, Dong-Yu Lv, Hong-Ji Li, Jin-Zhong Gu

**Affiliations:** a School of Chemistry and Biology Engineering, Taiyuan University of Science and Technology, Taiyuan 030021, People’s Republic of China; b Key Laboratory of Nonferrous Metal Chemistry and Resources Utilization of Gansu Province, College of Chemistry and Chemical Engineering, Lanzhou University, Lanzhou 730000, People’s Republic of China

## Abstract

In the title coordination polymer, [Nd_2_(C_10_H_8_O_4_)_3_(H_2_O)_2_]_*n*_, each of the two Nd^III^ ions is nine-coordinated by eight O atoms from six different 2,2′-(*m*-phenyl­ene)diacetate (pda) bivalent anions and by one O atom from a water mol­ecule, forming a distorted tricapped trigonal–prismatic coordination geometry. Eight Nd^III^ ions and 12 pda ligands form a large [Nd_8_(pda)_12_] ring, and four Nd^III^ ions and six pda ligands form a small [Nd_4_(pda)_6_] ring. These rings are further connected by the coordination inter­actions of pda ligands and Nd^III^, generating a three-dimensional supra­molecular framework.

## Related literature

For the isotypic Ce analogue, see: Gao *et al.* (2011 ▶). For the structures and properties of lanthanide coordination compounds, see: Xiao *et al.* (2008 ▶); Lv *et al.* (2010 ▶). For bond lengths and angles in other complexes with nine-coordinate Nd^III^, see: Xiao *et al.* (2008 ▶); Wang *et al.* (2009 ▶).
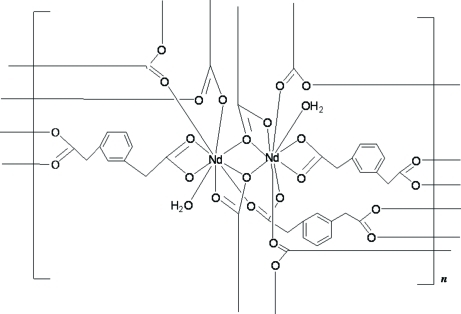


## Experimental

### 

#### Crystal data

[Nd_2_(C_10_H_8_O_4_)_3_(H_2_O)_2_]
*M* *_r_* = 901.00
Triclinic, 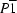

*a* = 10.4846 (13) Å
*b* = 11.9660 (16) Å
*c* = 12.3514 (16) Å
α = 105.619 (5)°
β = 97.202 (5)°
γ = 92.625 (6)°
*V* = 1475.5 (3) Å^3^
*Z* = 2
Mo *K*α radiation
μ = 3.55 mm^−1^
*T* = 296 K
0.24 × 0.22 × 0.20 mm

#### Data collection

Bruker SMART CCD diffractometer
Absorption correction: multi-scan (*SADABS*; Bruker, 1997 ▶) *T*_min_ = 0.516, *T*_max_ = 0.580
7865 measured reflections
5315 independent reflections
4806 reflections with *I* > 2σ(*I*)
*R*_int_ = 0.013

#### Refinement

*R*[*F* ^2^ > 2σ(*F* ^2^)] = 0.021
*wR*(*F* ^2^) = 0.054
*S* = 1.03
5315 reflections
415 parameters
3 restraints
H-atom parameters constrained
Δρ_max_ = 0.64 e Å^−3^
Δρ_min_ = −0.76 e Å^−3^

### 

Data collection: *SMART* (Bruker, 1997 ▶); cell refinement: *SAINT* (Bruker, 1997 ▶); data reduction: *SAINT*; program(s) used to solve structure: *SHELXS97* (Sheldrick, 2008 ▶); program(s) used to refine structure: *SHELXL97* (Sheldrick, 2008 ▶); molecular graphics: *XP* in *SHELXTL* (Sheldrick, 2008 ▶) and *DIAMOND* (Brandenburg, 2006 ▶); software used to prepare material for publication: *publCIF* (Westrip, 2010 ▶).

## Supplementary Material

Crystal structure: contains datablocks I, global. DOI: 10.1107/S1600536811006817/wm2461sup1.cif

Structure factors: contains datablocks I. DOI: 10.1107/S1600536811006817/wm2461Isup2.hkl

Additional supplementary materials:  crystallographic information; 3D view; checkCIF report

## Figures and Tables

**Table 1:** Hydrogen-bond geometry (Å, °)

*D*—H⋯*A*	*D*—H	H⋯*A*	*D*⋯*A*	*D*—H⋯*A*
O13—H1*W*⋯O12^i^	0.88	2.39	2.838 (3)	112
O14—H4*W*⋯O7^ii^	0.86	2.26	2.829 (3)	124
O14—H4*W*⋯O2^iii^	0.86	2.41	3.177 (4)	148
O14—H3*W*⋯O7	0.87	2.25	3.024 (3)	149
O14—H3*W*⋯O14^ii^	0.87	2.53	3.100 (5)	123

Symmetry codes: (i) 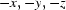; (ii) 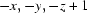; (iii) 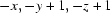.

**Fig. 1.**
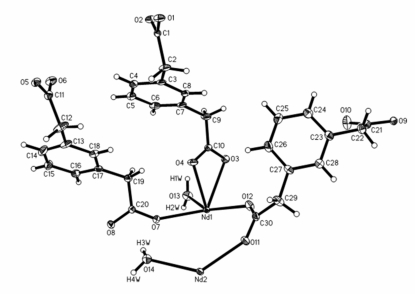
A drawing of the asymmetric unit in the structure of the title complex, showing the atom-labelling scheme. Displacement ellipsoids are drawn at the 30% probability level.

**Fig. 2.**
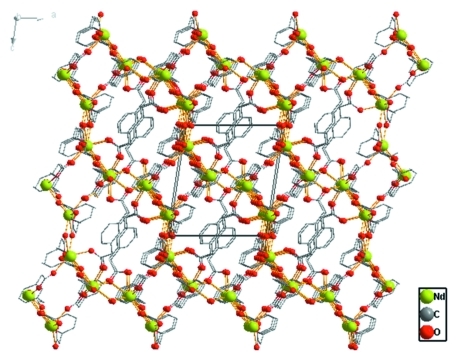
Unit cell packing of the title complex showing the three-dimensional framework formed by a large [Nd8(pda)12] ring and a small [Nd4(pda)6] ring.

## supplementary crystallographic information

### Comment

Lanthanide coordination polymers have shown versatile structural architectures, accompanied with desirable properties, like luminescence, magnetism, catalysis, gas adsorption and separation (Xiao *et al.*, 2008; Lv *et al.*, 2010). In order to extend our investigations in this field, we chose 1,3-phenylendiacetic acid (pda) as a functional ligand and synthesized the lanthanide coordination polymer [Nd_2_(pda)_3_(H_2_O)_2_]*_n_*, the structure of which is reported here.

The title compound is isotypic with its Ce analogue (Gao *et al.*, 2011). The asymmetric unit of the title complex (Fig. 1) contains two crystallographically unique Nd^III^ ions, three pda ligands, and two coordinated water molecules. Both Nd1 and Nd2 are nine-coordinated within a distorted tricapped trigonal-prismatic geometry. The nine coordination sites are occupied by one O atom from a water molecule and eight O atoms from six different pda anions. The Nd—O bond lengths in the title complex are in the range 2.377 (2)–2.749 (2) Å, which is comparable to those reported for other Nd complexes with oxygen environment around the central metal (Xiao *et al*., 2008; Wang *et al.*, 2009). The pda ligands adopt two coordination modes, *viz*. *µ*_4_-hexadentate and *µ*_4_-pentadentate. Eight Nd^III^ ions and twelve pda ligands form a large [Nd_8_(pda)_12_] ring, whereas four Nd^III^ ions and six pda ligands form a small [Nd_4_(pda)_6_] ring (Fig. 2). These rings are further connected by the coordination interactions of pda ligands and Nd^III^ to generate a three-dimensional supramolecular framework (Fig. 2).

### Experimental

To a solution of neodymium nitrate hexahydrate (0.088 g, 0.2 mmol) in water (5 ml) was added an aqueous solution (5 ml) of the ligand (0.058 g, 0.3 mmol) and a drop of triethylamine. The reactants were sealed in a 25-ml Teflon-lined stainless-steel Parr bomb. The bomb was heated at 433 K for 3 d. Upon cooling, the solution yielded single crystals of the title complex in *ca* 75% yield. Anal./calc. for C_30_H_28_Nd_2_O_14_: C, 39.99; H, 3.13; found: C, 40.43; H, 3.47.

### Refinement

The H atoms of the water molecules were located in a difference Fourier map and were refined with distance constraints of O–H = 0.83 (5) Å. The C-bound H atoms were placed in geometrically idealized positions, with C–H = 0.93 and 0.97 Å for aryl and methylene H-atoms, respectively, and constrained to ride on their respective parent atoms, with *U*_iso_(H) = 1.2 *U*_eq_(C).

### Crystal data

**Table:**

[Nd_2_(C_10_H_8_O_4_)_3_(H_2_O)_2_]	*Z* = 2
*M**_r_* = 901.00	*F*(000) = 880
Triclinic, *P*1	*D*_x_ = 2.028 Mg m^−^^3^
Hall symbol: -P 1	Mo *K*α radiation, λ = 0.71073 Å
*a* = 10.4846 (13) Å	Cell parameters from 5315 reflections
*b* = 11.9660 (16) Å	θ = 1.7–25.3°
*c* = 12.3514 (16) Å	µ = 3.55 mm^−^^1^
α = 105.619 (5)°	*T* = 296 K
β = 97.202 (5)°	Block, colorless
γ = 92.625 (6)°	0.24 × 0.22 × 0.20 mm
*V* = 1475.5 (3) Å^3^	

### Data collection

**Table:**

Bruker SMART CCD diffractometer	5315 independent reflections
Radiation source: fine-focus sealed tube	4806 reflections with *I* > 2σ(*I*)
graphite	*R*_int_ = 0.013
Detector resolution: 0 pixels mm^-1^	θ_max_ = 25.3°, θ_min_ = 1.7°
φ and ω scans	*h* = −12→12
Absorption correction: multi-scan (*SADABS*; Bruker, 1997)	*k* = −12→14
*T*_min_ = 0.516, *T*_max_ = 0.580	*l* = −14→14
7865 measured reflections	

### Refinement

**Table:**

Refinement on *F*^2^	Primary atom site location: structure-invariant direct methods
Least-squares matrix: full	Secondary atom site location: difference Fourier map
*R*[*F*^2^ > 2σ(*F*^2^)] = 0.021	Hydrogen site location: inferred from neighbouring sites
*wR*(*F*^2^) = 0.054	H-atom parameters constrained
*S* = 1.03	*w* = 1/[σ^2^(*F*_o_^2^) + (0.0265*P*)^2^ + 1.487*P*] where *P* = (*F*_o_^2^ + 2*F*_c_^2^)/3
5315 reflections	(Δ/σ)_max_ = 0.001
415 parameters	Δρ_max_ = 0.64 e Å^−^^3^
3 restraints	Δρ_min_ = −0.76 e Å^−^^3^

### Special details

**Table:**

Geometry. All e.s.d.'s (except the e.s.d. in the dihedral angle between two l.s. planes) are estimated using the full covariance matrix. The cell e.s.d.'s are taken into account individually in the estimation of e.s.d.'s in distances, angles and torsion angles; correlations between e.s.d.'s in cell parameters are only used when they are defined by crystal symmetry. An approximate (isotropic) treatment of cell e.s.d.'s is used for estimating e.s.d.'s involving l.s. planes.
Refinement. Refinement of *F*^2^ against ALL reflections. The weighted *R*-factor *wR* and goodness of fit *S* are based on *F*^2^, conventional *R*-factors *R* are based on *F*, with *F* set to zero for negative *F*^2^. The threshold expression of *F*^2^ > σ(*F*^2^) is used only for calculating *R*-factors(gt) *etc*. and is not relevant to the choice of reflections for refinement. *R*-factors based on *F*^2^ are statistically about twice as large as those based on *F*, and *R*- factors based on ALL data will be even larger.

### Fractional atomic coordinates and isotropic or equivalent isotropic displacement parameters (Å^2^)

**Table:**

	*x*	*y*	*z*	*U*_iso_*/*U*_eq_	
Nd1	0.029517 (16)	0.026539 (14)	0.183517 (13)	0.01602 (6)	
Nd2	0.306439 (16)	0.036922 (15)	0.455279 (14)	0.01818 (6)	
C1	0.0706 (3)	0.8190 (3)	0.2608 (3)	0.0194 (7)	
C2	0.1023 (4)	0.7042 (3)	0.2819 (3)	0.0314 (9)	
H2A	0.1766	0.6787	0.2453	0.038*	
H2B	0.1270	0.7169	0.3629	0.038*	
C3	−0.0041 (4)	0.6073 (3)	0.2411 (3)	0.0244 (8)	
C4	−0.1173 (4)	0.6146 (3)	0.2887 (3)	0.0340 (9)	
H4	−0.1275	0.6802	0.3468	0.041*	
C5	−0.2149 (4)	0.5267 (3)	0.2516 (4)	0.0368 (9)	
H5	−0.2897	0.5324	0.2853	0.044*	
C6	−0.2021 (4)	0.4299 (3)	0.1643 (3)	0.0330 (9)	
H6	−0.2691	0.3714	0.1383	0.040*	
C7	−0.0910 (4)	0.4192 (3)	0.1154 (3)	0.0265 (8)	
C8	0.0082 (4)	0.5078 (3)	0.1549 (3)	0.0252 (8)	
H8	0.0843	0.5002	0.1230	0.030*	
C9	−0.0755 (4)	0.3102 (3)	0.0240 (3)	0.0326 (9)	
H9A	−0.1557	0.2884	−0.0279	0.039*	
H9B	−0.0092	0.3272	−0.0188	0.039*	
C10	−0.0404 (3)	0.2088 (3)	0.0679 (3)	0.0220 (7)	
C11	−0.2153 (3)	0.7492 (3)	0.5878 (3)	0.0243 (7)	
C12	−0.1546 (4)	0.6428 (3)	0.6052 (4)	0.0475 (12)	
H12A	−0.0771	0.6353	0.5692	0.057*	
H12B	−0.1280	0.6558	0.6860	0.057*	
C13	−0.2359 (4)	0.5289 (3)	0.5614 (3)	0.0322 (9)	
C14	−0.3482 (4)	0.5093 (3)	0.6035 (3)	0.0393 (10)	
H14	−0.3755	0.5681	0.6597	0.047*	
C15	−0.4198 (4)	0.4044 (3)	0.5636 (3)	0.0334 (9)	
H15	−0.4955	0.3927	0.5924	0.040*	
C16	−0.3801 (3)	0.3155 (3)	0.4805 (3)	0.0280 (8)	
H16	−0.4301	0.2449	0.4526	0.034*	
C17	−0.2661 (3)	0.3315 (3)	0.4388 (3)	0.0232 (7)	
C18	−0.1952 (3)	0.4387 (3)	0.4794 (3)	0.0258 (8)	
H18	−0.1190	0.4506	0.4512	0.031*	
C19	−0.2165 (3)	0.2365 (3)	0.3508 (3)	0.0257 (8)	
H19A	−0.1419	0.2690	0.3271	0.031*	
H19B	−0.2825	0.2110	0.2848	0.031*	
C20	−0.1792 (3)	0.1314 (3)	0.3893 (3)	0.0194 (7)	
C21	0.4696 (3)	0.1360 (3)	−0.3232 (3)	0.0249 (8)	
C22	0.5520 (3)	0.2052 (3)	−0.2133 (3)	0.0289 (8)	
H22A	0.6237	0.1609	−0.1973	0.035*	
H22B	0.5873	0.2772	−0.2238	0.035*	
C23	0.4804 (3)	0.2339 (3)	−0.1121 (3)	0.0272 (8)	
C24	0.3884 (5)	0.3138 (4)	−0.1054 (4)	0.0496 (12)	
H24	0.3733	0.3507	−0.1626	0.060*	
H1W	−0.2406	0.0218	0.0635	0.060*	
H2W	−0.2406	−0.0802	0.1125	0.060*	
H4W	0.1094	0.0990	0.6110	0.060*	
H3W	0.0294	0.0896	0.5035	0.060*	
C25	0.3193 (5)	0.3397 (4)	−0.0164 (4)	0.0574 (14)	
H25	0.2561	0.3919	−0.0146	0.069*	
C26	0.3432 (4)	0.2885 (4)	0.0707 (3)	0.0409 (10)	
H26	0.2972	0.3075	0.1319	0.049*	
C27	0.4349 (3)	0.2093 (3)	0.0676 (3)	0.0270 (8)	
C28	0.5025 (3)	0.1825 (3)	−0.0248 (3)	0.0261 (8)	
H28	0.5641	0.1286	−0.0278	0.031*	
C29	0.4652 (4)	0.1561 (4)	0.1644 (3)	0.0348 (9)	
H29A	0.5015	0.2183	0.2310	0.042*	
H29B	0.5324	0.1040	0.1459	0.042*	
C30	0.3579 (3)	0.0892 (3)	0.1978 (3)	0.0242 (7)	
O1	0.1566 (2)	0.9053 (2)	0.2968 (2)	0.0274 (5)	
O2	−0.0342 (2)	0.8302 (2)	0.2080 (2)	0.0284 (6)	
O3	−0.0371 (3)	0.10987 (19)	−0.0009 (2)	0.0300 (6)	
O4	−0.0146 (3)	0.2218 (2)	0.1716 (2)	0.0346 (6)	
O5	−0.1595 (2)	0.84733 (19)	0.64899 (19)	0.0252 (5)	
O6	−0.3093 (2)	0.7452 (2)	0.5148 (2)	0.0312 (6)	
O7	−0.0998 (2)	0.0686 (2)	0.3388 (2)	0.0274 (5)	
O8	−0.2271 (3)	0.1114 (2)	0.4695 (2)	0.0365 (6)	
O9	0.5273 (2)	0.1006 (2)	−0.41110 (18)	0.0243 (5)	
O10	0.3524 (3)	0.1179 (3)	−0.3299 (2)	0.0445 (7)	
O11	0.3881 (2)	0.0619 (2)	0.2879 (2)	0.0300 (6)	
O12	0.2492 (2)	0.0633 (2)	0.1359 (2)	0.0308 (6)	
O13	−0.2057 (2)	−0.0221 (2)	0.1036 (2)	0.0355 (6)	
O14	0.1067 (2)	0.1022 (2)	0.5416 (2)	0.0334 (6)	

### Atomic displacement parameters (Å^2^)

**Table:**

	*U*^11^	*U*^22^	*U*^33^	*U*^12^	*U*^13^	*U*^23^
Nd1	0.02272 (10)	0.01120 (9)	0.01384 (9)	0.00192 (7)	0.00258 (7)	0.00292 (7)
Nd2	0.02183 (10)	0.01863 (10)	0.01494 (10)	0.00167 (7)	0.00283 (7)	0.00602 (7)
C1	0.0306 (18)	0.0139 (16)	0.0136 (15)	0.0028 (14)	0.0065 (14)	0.0017 (13)
C2	0.043 (2)	0.0156 (18)	0.037 (2)	0.0046 (15)	−0.0013 (17)	0.0105 (16)
C3	0.038 (2)	0.0119 (16)	0.0242 (18)	0.0036 (14)	0.0010 (15)	0.0084 (14)
C4	0.060 (3)	0.0161 (18)	0.029 (2)	0.0100 (17)	0.0159 (19)	0.0066 (15)
C5	0.044 (2)	0.030 (2)	0.046 (2)	0.0107 (18)	0.0183 (19)	0.0189 (19)
C6	0.041 (2)	0.0188 (19)	0.040 (2)	−0.0011 (16)	−0.0013 (18)	0.0131 (17)
C7	0.045 (2)	0.0113 (17)	0.0229 (18)	0.0071 (15)	−0.0007 (16)	0.0063 (14)
C8	0.037 (2)	0.0177 (17)	0.0242 (17)	0.0095 (15)	0.0074 (15)	0.0093 (14)
C9	0.057 (3)	0.0178 (18)	0.0218 (18)	0.0051 (17)	0.0011 (17)	0.0055 (15)
C10	0.0297 (18)	0.0129 (17)	0.0227 (18)	0.0016 (13)	0.0054 (14)	0.0032 (14)
C11	0.0265 (18)	0.0175 (18)	0.0254 (18)	−0.0028 (14)	0.0044 (15)	0.0005 (14)
C12	0.039 (2)	0.016 (2)	0.073 (3)	0.0030 (17)	−0.018 (2)	−0.0002 (19)
C13	0.034 (2)	0.0155 (18)	0.041 (2)	0.0040 (15)	−0.0102 (17)	0.0048 (16)
C14	0.048 (3)	0.029 (2)	0.036 (2)	0.0154 (19)	0.0028 (19)	−0.0006 (17)
C15	0.032 (2)	0.030 (2)	0.039 (2)	0.0066 (16)	0.0131 (17)	0.0079 (17)
C16	0.0314 (19)	0.0191 (18)	0.033 (2)	0.0000 (15)	0.0033 (16)	0.0077 (15)
C17	0.0307 (19)	0.0192 (17)	0.0210 (17)	0.0042 (14)	0.0007 (14)	0.0087 (14)
C18	0.0224 (17)	0.0199 (18)	0.035 (2)	0.0017 (14)	−0.0010 (15)	0.0103 (15)
C19	0.0315 (19)	0.0219 (18)	0.0252 (18)	0.0034 (15)	0.0049 (15)	0.0082 (15)
C20	0.0236 (17)	0.0151 (16)	0.0167 (16)	−0.0015 (13)	0.0018 (13)	0.0007 (13)
C21	0.0294 (19)	0.030 (2)	0.0158 (17)	0.0037 (15)	0.0031 (14)	0.0064 (15)
C22	0.0305 (19)	0.035 (2)	0.0192 (18)	0.0000 (16)	0.0027 (15)	0.0038 (16)
C23	0.033 (2)	0.030 (2)	0.0161 (17)	−0.0001 (15)	0.0021 (15)	0.0036 (15)
C24	0.069 (3)	0.055 (3)	0.039 (2)	0.029 (2)	0.026 (2)	0.026 (2)
C25	0.077 (3)	0.063 (3)	0.050 (3)	0.043 (3)	0.037 (3)	0.027 (2)
C26	0.056 (3)	0.040 (2)	0.030 (2)	0.010 (2)	0.0212 (19)	0.0081 (18)
C27	0.0319 (19)	0.0269 (19)	0.0227 (18)	−0.0050 (15)	0.0032 (15)	0.0094 (15)
C28	0.0246 (18)	0.0267 (19)	0.0253 (18)	−0.0011 (14)	0.0007 (15)	0.0060 (15)
C29	0.030 (2)	0.047 (2)	0.030 (2)	−0.0072 (17)	0.0004 (16)	0.0188 (18)
C30	0.0282 (19)	0.0257 (19)	0.0193 (17)	0.0035 (15)	0.0082 (15)	0.0046 (15)
O1	0.0361 (14)	0.0165 (12)	0.0282 (13)	−0.0012 (10)	−0.0038 (11)	0.0079 (10)
O2	0.0297 (13)	0.0205 (13)	0.0363 (14)	0.0001 (10)	−0.0006 (11)	0.0126 (11)
O3	0.0495 (16)	0.0138 (12)	0.0258 (13)	0.0040 (11)	0.0117 (12)	0.0010 (10)
O4	0.0642 (19)	0.0190 (13)	0.0199 (13)	0.0106 (12)	−0.0007 (12)	0.0060 (10)
O5	0.0326 (13)	0.0158 (12)	0.0228 (12)	0.0005 (10)	−0.0011 (10)	0.0001 (10)
O6	0.0322 (14)	0.0198 (13)	0.0356 (14)	−0.0023 (10)	−0.0064 (12)	0.0035 (11)
O7	0.0340 (14)	0.0250 (13)	0.0264 (13)	0.0103 (11)	0.0121 (11)	0.0080 (11)
O8	0.0593 (18)	0.0261 (14)	0.0353 (15)	0.0142 (13)	0.0287 (14)	0.0160 (12)
O9	0.0316 (13)	0.0260 (13)	0.0162 (11)	0.0074 (10)	0.0064 (10)	0.0050 (10)
O10	0.0263 (15)	0.073 (2)	0.0265 (14)	−0.0010 (14)	0.0021 (12)	0.0016 (14)
O11	0.0310 (14)	0.0403 (15)	0.0247 (13)	0.0063 (11)	0.0083 (11)	0.0165 (12)
O12	0.0262 (13)	0.0424 (16)	0.0236 (13)	−0.0044 (11)	0.0031 (11)	0.0100 (12)
O13	0.0298 (14)	0.0386 (16)	0.0362 (15)	−0.0043 (12)	−0.0032 (12)	0.0119 (12)
O14	0.0334 (14)	0.0384 (16)	0.0316 (14)	0.0090 (12)	0.0078 (11)	0.0129 (12)

### Geometric parameters (Å, °)

**Table:**

Nd1—O3^i^	2.415 (2)	C15—C16	1.384 (5)
Nd1—O5^ii^	2.416 (2)	C15—H15	0.9300
Nd1—O4	2.441 (2)	C16—C17	1.384 (5)
Nd1—O7	2.442 (2)	C16—H16	0.9300
Nd1—O12	2.496 (2)	C17—C18	1.387 (5)
Nd1—O13	2.519 (2)	C17—C19	1.508 (5)
Nd1—O2^iii^	2.520 (2)	C18—H18	0.9300
Nd1—O1^iii^	2.576 (2)	C19—C20	1.510 (5)
Nd1—O3	2.749 (2)	C19—H19A	0.9700
Nd2—O8^iv^	2.377 (2)	C19—H19B	0.9700
Nd2—O11	2.418 (2)	C20—O8	1.239 (4)
Nd2—O9^v^	2.462 (2)	C20—O7	1.257 (4)
Nd2—O1^iii^	2.475 (2)	C21—O10	1.227 (4)
Nd2—O14	2.529 (3)	C21—O9	1.290 (4)
Nd2—O6^ii^	2.531 (2)	C21—C22	1.519 (5)
Nd2—O10^vi^	2.542 (3)	C21—Nd2^vii^	2.952 (3)
Nd2—O5^ii^	2.571 (2)	C22—C23	1.506 (5)
Nd2—O9^vi^	2.621 (2)	C22—H22A	0.9700
C1—O2	1.237 (4)	C22—H22B	0.9700
C1—O1	1.282 (4)	C23—C28	1.380 (5)
C1—C2	1.510 (4)	C23—C24	1.383 (5)
C2—C3	1.506 (5)	C24—C25	1.366 (6)
C2—H2A	0.9700	C24—H24	0.9300
C2—H2B	0.9700	C25—C26	1.377 (6)
C3—C4	1.385 (5)	C25—H25	0.9300
C3—C8	1.392 (5)	C26—C27	1.377 (5)
C4—C5	1.374 (6)	C26—H26	0.9300
C4—H4	0.9300	C27—C28	1.391 (5)
C5—C6	1.378 (5)	C27—C29	1.507 (5)
C5—H5	0.9300	C28—H28	0.9300
C6—C7	1.373 (5)	C29—C30	1.512 (5)
C6—H6	0.9300	C29—H29A	0.9700
C7—C8	1.392 (5)	C29—H29B	0.9700
C7—C9	1.508 (5)	C30—O11	1.251 (4)
C8—H8	0.9300	C30—O12	1.266 (4)
C9—C10	1.499 (5)	O1—Nd2^viii^	2.475 (2)
C9—H9A	0.9700	O1—Nd1^viii^	2.576 (2)
C9—H9B	0.9700	O2—Nd1^viii^	2.520 (2)
C10—O4	1.241 (4)	O3—Nd1^i^	2.415 (2)
C10—O3	1.263 (4)	O5—Nd1^ii^	2.416 (2)
C11—O6	1.239 (4)	O5—Nd2^ii^	2.571 (2)
C11—O5	1.281 (4)	O6—Nd2^ii^	2.531 (2)
C11—C12	1.503 (5)	O8—Nd2^iv^	2.377 (2)
C12—C13	1.502 (5)	O9—Nd2^v^	2.462 (2)
C12—H12A	0.9700	O9—Nd2^vii^	2.621 (2)
C12—H12B	0.9700	O10—Nd2^vii^	2.542 (3)
C13—C14	1.381 (6)	O13—H1W	0.8758
C13—C18	1.391 (5)	O13—H2W	0.8110
C14—C15	1.367 (6)	O14—H4W	0.8644
C14—H14	0.9300	O14—H3W	0.8693
			
O3^i^—Nd1—O5^ii^	144.23 (9)	C7—C9—H9B	108.7
O3^i^—Nd1—O4	113.33 (8)	H9A—C9—H9B	107.6
O5^ii^—Nd1—O4	76.46 (8)	O4—C10—O3	119.9 (3)
O3^i^—Nd1—O7	141.44 (8)	O4—C10—C9	120.3 (3)
O5^ii^—Nd1—O7	71.16 (8)	O3—C10—C9	119.9 (3)
O4—Nd1—O7	84.45 (8)	O6—C11—O5	120.5 (3)
O3^i^—Nd1—O12	74.32 (8)	O6—C11—C12	123.3 (3)
O5^ii^—Nd1—O12	71.68 (8)	O5—C11—C12	116.0 (3)
O4—Nd1—O12	88.05 (9)	C13—C12—C11	117.0 (3)
O7—Nd1—O12	142.83 (8)	C13—C12—H12A	108.0
O3^i^—Nd1—O13	77.43 (9)	C11—C12—H12A	108.0
O5^ii^—Nd1—O13	138.26 (8)	C13—C12—H12B	108.0
O4—Nd1—O13	83.71 (9)	C11—C12—H12B	108.0
O7—Nd1—O13	70.65 (8)	H12A—C12—H12B	107.3
O12—Nd1—O13	144.46 (8)	C14—C13—C18	118.6 (3)
O3^i^—Nd1—O2^iii^	75.03 (8)	C14—C13—C12	121.8 (4)
O5^ii^—Nd1—O2^iii^	112.51 (8)	C18—C13—C12	119.6 (4)
O4—Nd1—O2^iii^	153.02 (9)	C15—C14—C13	120.8 (4)
O7—Nd1—O2^iii^	75.35 (8)	C15—C14—H14	119.6
O12—Nd1—O2^iii^	118.83 (8)	C13—C14—H14	119.6
O13—Nd1—O2^iii^	72.90 (8)	C14—C15—C16	120.4 (4)
O3^i^—Nd1—O1^iii^	94.43 (8)	C14—C15—H15	119.8
O5^ii^—Nd1—O1^iii^	69.57 (8)	C16—C15—H15	119.8
O4—Nd1—O1^iii^	146.02 (8)	C15—C16—C17	120.1 (3)
O7—Nd1—O1^iii^	85.44 (8)	C15—C16—H16	119.9
O12—Nd1—O1^iii^	80.76 (8)	C17—C16—H16	119.9
O13—Nd1—O1^iii^	122.85 (8)	C16—C17—C18	118.8 (3)
O2^iii^—Nd1—O1^iii^	50.71 (7)	C16—C17—C19	122.3 (3)
O3^i^—Nd1—O3	64.78 (9)	C18—C17—C19	118.9 (3)
O5^ii^—Nd1—O3	119.11 (7)	C17—C18—C13	121.2 (3)
O4—Nd1—O3	48.91 (7)	C17—C18—H18	119.4
O7—Nd1—O3	119.10 (8)	C13—C18—H18	119.4
O12—Nd1—O3	80.67 (8)	C17—C19—C20	115.1 (3)
O13—Nd1—O3	67.92 (8)	C17—C19—H19A	108.5
O2^iii^—Nd1—O3	128.34 (7)	C20—C19—H19A	108.5
O1^iii^—Nd1—O3	155.25 (8)	C17—C19—H19B	108.5
O8^iv^—Nd2—O11	140.87 (9)	C20—C19—H19B	108.5
O8^iv^—Nd2—O9^v^	80.82 (8)	H19A—C19—H19B	107.5
O11—Nd2—O9^v^	72.33 (8)	O8—C20—O7	123.0 (3)
O8^iv^—Nd2—O1^iii^	74.90 (9)	O8—C20—C19	118.7 (3)
O11—Nd2—O1^iii^	76.48 (8)	O7—C20—C19	118.3 (3)
O9^v^—Nd2—O1^iii^	88.60 (8)	O10—C21—O9	120.9 (3)
O8^iv^—Nd2—O14	71.83 (9)	O10—C21—C22	121.8 (3)
O11—Nd2—O14	131.77 (8)	O9—C21—C22	117.3 (3)
O9^v^—Nd2—O14	152.59 (8)	C23—C22—C21	114.1 (3)
O1^iii^—Nd2—O14	85.98 (8)	C23—C22—H22A	108.7
O8^iv^—Nd2—O6^ii^	140.58 (9)	C21—C22—H22A	108.7
O11—Nd2—O6^ii^	77.47 (9)	C23—C22—H22B	108.7
O9^v^—Nd2—O6^ii^	132.09 (8)	C21—C22—H22B	108.7
O1^iii^—Nd2—O6^ii^	119.41 (8)	H22A—C22—H22B	107.6
O14—Nd2—O6^ii^	72.89 (9)	C28—C23—C24	118.0 (3)
O8^iv^—Nd2—O10^vi^	73.99 (10)	C28—C23—C22	122.3 (3)
O11—Nd2—O10^vi^	139.31 (9)	C24—C23—C22	119.7 (3)
O9^v^—Nd2—O10^vi^	103.61 (8)	C25—C24—C23	121.3 (4)
O1^iii^—Nd2—O10^vi^	144.10 (9)	C25—C24—H24	119.4
O14—Nd2—O10^vi^	67.60 (8)	C23—C24—H24	119.4
O6^ii^—Nd2—O10^vi^	76.78 (9)	C24—C25—C26	120.1 (4)
O8^iv^—Nd2—O5^ii^	123.39 (9)	C24—C25—H25	120.0
O11—Nd2—O5^ii^	67.91 (8)	C26—C25—H25	120.0
O9^v^—Nd2—O5^ii^	137.67 (7)	C25—C26—C27	120.4 (4)
O1^iii^—Nd2—O5^ii^	68.79 (7)	C25—C26—H26	119.8
O14—Nd2—O5^ii^	63.87 (8)	C27—C26—H26	119.8
O6^ii^—Nd2—O5^ii^	50.79 (7)	C26—C27—C28	118.7 (3)
O10^vi^—Nd2—O5^ii^	115.78 (9)	C26—C27—C29	120.8 (3)
O8^iv^—Nd2—O9^vi^	99.49 (9)	C28—C27—C29	120.5 (3)
O11—Nd2—O9^vi^	94.98 (8)	C23—C28—C27	121.6 (3)
O9^v^—Nd2—O9^vi^	65.88 (9)	C23—C28—H28	119.2
O1^iii^—Nd2—O9^vi^	154.47 (8)	C27—C28—H28	119.2
O14—Nd2—O9^vi^	116.39 (8)	C27—C29—C30	119.0 (3)
O6^ii^—Nd2—O9^vi^	80.98 (7)	C27—C29—H29A	107.6
O10^vi^—Nd2—O9^vi^	50.17 (8)	C30—C29—H29A	107.6
O5^ii^—Nd2—O9^vi^	130.65 (7)	C27—C29—H29B	107.6
O2—C1—O1	120.1 (3)	C30—C29—H29B	107.6
O2—C1—C2	121.6 (3)	H29A—C29—H29B	107.0
O1—C1—C2	118.3 (3)	O11—C30—O12	125.0 (3)
C3—C2—C1	115.8 (3)	O11—C30—C29	114.2 (3)
C3—C2—H2A	108.3	O12—C30—C29	120.8 (3)
C1—C2—H2A	108.3	C1—O1—Nd2^viii^	149.4 (2)
C3—C2—H2B	108.3	C1—O1—Nd1^viii^	92.21 (19)
C1—C2—H2B	108.3	Nd2^viii^—O1—Nd1^viii^	109.46 (8)
H2A—C2—H2B	107.4	C1—O2—Nd1^viii^	96.0 (2)
C4—C3—C8	118.0 (3)	C10—O3—Nd1^i^	155.9 (2)
C4—C3—C2	120.9 (3)	C10—O3—Nd1	87.89 (19)
C8—C3—C2	121.1 (3)	Nd1^i^—O3—Nd1	115.22 (9)
C5—C4—C3	121.1 (4)	C10—O4—Nd1	103.32 (19)
C5—C4—H4	119.4	C11—O5—Nd1^ii^	153.8 (2)
C3—C4—H4	119.4	C11—O5—Nd2^ii^	92.8 (2)
C4—C5—C6	120.1 (4)	Nd1^ii^—O5—Nd2^ii^	111.55 (9)
C4—C5—H5	120.0	C11—O6—Nd2^ii^	95.8 (2)
C6—C5—H5	120.0	C20—O7—Nd1	147.9 (2)
C7—C6—C5	120.5 (4)	C20—O8—Nd2^iv^	144.9 (2)
C7—C6—H6	119.7	C21—O9—Nd2^v^	132.8 (2)
C5—C6—H6	119.7	C21—O9—Nd2^vii^	91.54 (19)
C6—C7—C8	119.0 (3)	Nd2^v^—O9—Nd2^vii^	114.12 (8)
C6—C7—C9	120.0 (3)	C21—O10—Nd2^vii^	96.9 (2)
C8—C7—C9	120.9 (3)	C30—O11—Nd2	143.5 (2)
C7—C8—C3	121.3 (3)	C30—O12—Nd1	131.1 (2)
C7—C8—H8	119.4	Nd1—O13—H1W	116.9
C3—C8—H8	119.4	Nd1—O13—H2W	117.1
C10—C9—C7	114.1 (3)	H1W—O13—H2W	125.7
C10—C9—H9A	108.7	Nd2—O14—H4W	113.5
C7—C9—H9A	108.7	Nd2—O14—H3W	123.7
C10—C9—H9B	108.7	H4W—O14—H3W	113.8
			
O2—C1—C2—C3	−3.6 (5)	O1^iii^—Nd1—O3—C10	−137.8 (2)
O1—C1—C2—C3	178.0 (3)	C1^iii^—Nd1—O3—C10	171.40 (19)
C1—C2—C3—C4	−65.1 (5)	O3^i^—Nd1—O3—Nd1^i^	0.0
C1—C2—C3—C8	115.0 (4)	O5^ii^—Nd1—O3—Nd1^i^	139.84 (10)
C8—C3—C4—C5	−0.4 (5)	O4—Nd1—O3—Nd1^i^	172.57 (17)
C2—C3—C4—C5	179.8 (3)	O7—Nd1—O3—Nd1^i^	−136.64 (10)
C3—C4—C5—C6	−1.1 (6)	O12—Nd1—O3—Nd1^i^	76.98 (11)
C4—C5—C6—C7	1.4 (6)	O13—Nd1—O3—Nd1^i^	−86.07 (11)
C5—C6—C7—C8	−0.2 (5)	O2^iii^—Nd1—O3—Nd1^i^	−42.57 (15)
C5—C6—C7—C9	176.9 (3)	O1^iii^—Nd1—O3—Nd1^i^	35.2 (2)
C6—C7—C8—C3	−1.3 (5)	C1^iii^—Nd1—O3—Nd1^i^	−15.6 (2)
C9—C7—C8—C3	−178.4 (3)	O3—C10—O4—Nd1	−0.8 (4)
C4—C3—C8—C7	1.6 (5)	C9—C10—O4—Nd1	179.2 (3)
C2—C3—C8—C7	−178.6 (3)	O3^i^—Nd1—O4—C10	7.8 (3)
C6—C7—C9—C10	−78.4 (4)	O5^ii^—Nd1—O4—C10	151.4 (2)
C8—C7—C9—C10	98.7 (4)	O7—Nd1—O4—C10	−136.7 (2)
C7—C9—C10—O4	−6.9 (5)	O12—Nd1—O4—C10	79.8 (2)
C7—C9—C10—O3	173.1 (3)	O13—Nd1—O4—C10	−65.6 (2)
O6—C11—C12—C13	−21.0 (6)	O2^iii^—Nd1—O4—C10	−95.4 (3)
O5—C11—C12—C13	162.9 (4)	O1^iii^—Nd1—O4—C10	150.0 (2)
C11—C12—C13—C14	−62.9 (6)	O3—Nd1—O4—C10	0.4 (2)
C11—C12—C13—C18	119.6 (4)	C1^iii^—Nd1—O4—C10	−162.0 (3)
C18—C13—C14—C15	−1.6 (6)	O6—C11—O5—Nd1^ii^	156.4 (4)
C12—C13—C14—C15	−179.1 (4)	C12—C11—O5—Nd1^ii^	−27.5 (7)
C13—C14—C15—C16	0.5 (6)	O6—C11—O5—Nd2^ii^	−2.8 (3)
C14—C15—C16—C17	1.3 (6)	C12—C11—O5—Nd2^ii^	173.3 (3)
C15—C16—C17—C18	−1.9 (5)	O5—C11—O6—Nd2^ii^	2.9 (4)
C15—C16—C17—C19	178.2 (3)	C12—C11—O6—Nd2^ii^	−173.0 (4)
C16—C17—C18—C13	0.8 (5)	O8—C20—O7—Nd1	175.1 (3)
C19—C17—C18—C13	−179.3 (3)	C19—C20—O7—Nd1	−5.4 (6)
C14—C13—C18—C17	0.9 (5)	O3^i^—Nd1—O7—C20	−105.2 (4)
C12—C13—C18—C17	178.5 (3)	O5^ii^—Nd1—O7—C20	93.5 (4)
C16—C17—C19—C20	−65.2 (4)	O4—Nd1—O7—C20	15.9 (4)
C18—C17—C19—C20	114.9 (4)	O12—Nd1—O7—C20	95.2 (4)
C17—C19—C20—O8	22.9 (5)	O13—Nd1—O7—C20	−69.4 (4)
C17—C19—C20—O7	−156.6 (3)	O2^iii^—Nd1—O7—C20	−146.1 (4)
O10—C21—C22—C23	8.0 (5)	O1^iii^—Nd1—O7—C20	163.4 (4)
O9—C21—C22—C23	−174.6 (3)	O3—Nd1—O7—C20	−20.0 (4)
C21—C22—C23—C28	109.6 (4)	C1^iii^—Nd1—O7—C20	−170.7 (4)
C21—C22—C23—C24	−70.0 (5)	O7—C20—O8—Nd2^iv^	−36.4 (6)
C28—C23—C24—C25	−1.3 (7)	C19—C20—O8—Nd2^iv^	144.1 (3)
C22—C23—C24—C25	178.3 (4)	O10—C21—O9—Nd2^v^	−118.1 (3)
C23—C24—C25—C26	2.0 (8)	C22—C21—O9—Nd2^v^	64.4 (4)
C24—C25—C26—C27	−1.3 (8)	O10—C21—O9—Nd2^vii^	7.5 (4)
C25—C26—C27—C28	0.1 (6)	C22—C21—O9—Nd2^vii^	−170.0 (3)
C25—C26—C27—C29	177.8 (4)	O9—C21—O10—Nd2^vii^	−7.8 (4)
C24—C23—C28—C27	0.1 (6)	C22—C21—O10—Nd2^vii^	169.6 (3)
C22—C23—C28—C27	−179.5 (3)	O12—C30—O11—Nd2	−36.9 (6)
C26—C27—C28—C23	0.5 (5)	C29—C30—O11—Nd2	144.4 (3)
C29—C27—C28—C23	−177.2 (3)	O8^iv^—Nd2—O11—C30	100.6 (4)
C26—C27—C29—C30	58.1 (5)	O9^v^—Nd2—O11—C30	149.5 (4)
C28—C27—C29—C30	−124.2 (4)	O1^iii^—Nd2—O11—C30	56.7 (4)
C27—C29—C30—O11	−170.9 (3)	O14—Nd2—O11—C30	−15.2 (4)
C27—C29—C30—O12	10.4 (5)	O6^ii^—Nd2—O11—C30	−68.1 (4)
O2—C1—O1—Nd2^viii^	126.3 (4)	O10^vi^—Nd2—O11—C30	−120.0 (4)
C2—C1—O1—Nd2^viii^	−55.3 (6)	O5^ii^—Nd2—O11—C30	−15.6 (4)
O2—C1—O1—Nd1^viii^	−9.9 (3)	O9^vi^—Nd2—O11—C30	−147.7 (4)
C2—C1—O1—Nd1^viii^	168.6 (3)	C11^ii^—Nd2—O11—C30	−43.0 (4)
O1—C1—O2—Nd1^viii^	10.1 (3)	C21^vi^—Nd2—O11—C30	−134.6 (4)
C2—C1—O2—Nd1^viii^	−168.2 (3)	O11—C30—O12—Nd1	27.9 (5)
O4—C10—O3—Nd1^i^	−163.6 (4)	C29—C30—O12—Nd1	−153.5 (3)
C9—C10—O3—Nd1^i^	16.4 (8)	O3^i^—Nd1—O12—C30	−142.6 (3)
O4—C10—O3—Nd1	0.7 (3)	O5^ii^—Nd1—O12—C30	26.1 (3)
C9—C10—O3—Nd1	−179.3 (3)	O4—Nd1—O12—C30	102.4 (3)
O3^i^—Nd1—O3—C10	−173.0 (3)	O7—Nd1—O12—C30	24.3 (4)
O5^ii^—Nd1—O3—C10	−33.2 (2)	O13—Nd1—O12—C30	178.8 (3)
O4—Nd1—O3—C10	−0.43 (19)	O2^iii^—Nd1—O12—C30	−80.1 (3)
O7—Nd1—O3—C10	50.4 (2)	O1^iii^—Nd1—O12—C30	−45.4 (3)
O12—Nd1—O3—C10	−96.0 (2)	O3—Nd1—O12—C30	151.1 (3)
O13—Nd1—O3—C10	100.9 (2)	C1^iii^—Nd1—O12—C30	−63.8 (3)
O2^iii^—Nd1—O3—C10	144.43 (19)		

Symmetry codes: (i) −*x*, −*y*, −*z*; (ii) −*x*, −*y*+1, −*z*+1; (iii) *x*, *y*−1, *z*; (iv) −*x*, −*y*, −*z*+1; (v) −*x*+1, −*y*, −*z*; (vi) *x*, *y*, *z*+1; (vii) *x*, *y*, *z*−1; (viii) *x*, *y*+1, *z*.

### Hydrogen-bond geometry (Å, °)

**Table:**

*D*—H···*A*	*D*—H	H···*A*	*D*···*A*	*D*—H···*A*
O13—H1W···O12^i^	0.88	2.39	2.838 (3)	112
O14—H4W···O7^iv^	0.86	2.26	2.829 (3)	124
O14—H4W···O2^ii^	0.86	2.41	3.177 (4)	148
O14—H3W···O7	0.87	2.25	3.024 (3)	149
O14—H3W···O14^iv^	0.87	2.53	3.100 (5)	123

Symmetry codes: (i) −*x*, −*y*, −*z*; (iv) −*x*, −*y*, −*z*+1; (ii) −*x*, −*y*+1, −*z*+1.
